# Characterization and performance of a toluene-degrading biofilm developed on pumice stones

**DOI:** 10.1186/1475-2859-4-4

**Published:** 2005-01-17

**Authors:** Alessandra Di Lorenzo, Mario Varcamonti, Palma Parascandola, Rodolfo Vignola, Adriano Bernardi, Pasquale Sacceddu, Raffaello Sisto, Elisabetta de Alteriis

**Affiliations:** 1 Dip.to Fisiologia Generale ed Ambientale, Sez. Igiene e Microbiologia, Università degli Studi "Federico II", Naples, Italy; 2 Dip.to Ingegneria Chimica e Alimentare, Università di Salerno, Fisciano, Salerno, Italy; 3 EniTecnologie S.p.A, Monterotondo, Rome, Italy

## Abstract

**Background:**

Hydrocarbon-degrading biofilms in the treatment of contaminated groundwaters have received increasing attention due to the role played in the so-called "biobarriers". These are bioremediation systems in which a microbial consortium adherent to a solid support is placed across the flow of a contaminated plume, thus promoting biodegradation of the pollutant.

**Results:**

A microbial consortium adherent to pumice granules (biofilm) developed from a toluene-enriched microflora in a mini-scale system, following continuous supply of a mineral medium containing toluene, over a 12-month period. Observation by scanning electron microscopy, together with quantification of the biomass attached to pumice, evidenced the presence of abundant exopolymeric material surrounding the cells in the biofilm. Toluene removal monitored during 12-month operation, reached 99%. Identification of the species, based on comparative 16S ribosomal DNA (rDNA) sequence analysis, revealed that *Rhodococcus erythropolis *and *Pseudomonas marginalis *were the predominant bacterial species in the microbial consortium.

**Conclusion:**

A structurally complex toluene-degrading biofilm, mainly formed by *Rhodococcus erythropolis *and *Pseudomonas marginalis*, developed on pumice granules, in a mini-scale apparatus continuously fed with toluene.

## Background

Surface-attached microbial communities, known as biofilms, are traditionally employed in fixed-film reactors for wastewater treatment. More recently, biofilms originating from the indigenous microflora of a contaminated groundwater have received increasing attention due to the possibility to develop *in situ *bioremediation systems, directly placed across the flow of a contaminated plume, the so-called "biobarriers" [1]. These are particularly attractive in the case of hydrocarbon contaminated groundwater, since the target contaminants can be destroyed by the attached biomass, leaving potentially non-toxic chemicals as biodegradation products.

Indeed, the reactive system of a biobarrier is represented by the biofilm developed on the solid support starting from the autochtonous microbial population of the groundwater, where potentially degrading species are present.

In this communication, we report the characterization of a toluene-degrading biofilm developed on pumice granules in two packing mini-columns, employed as a laboratory-scale biobarrier, over a 12-month period. That time was long enough for a mature consortium to establish on a solid support [2].

The main objective of the work was to analyse the structure and composition of the biofilm established on pumice stones in a mini-scale apparatus and to ascertain its degradative capability towards toluene, as a starting point for potential applications of a pumice-bound microbial consortium in bioremediation. Pumice, a rock of volcanic origin very abundant in Southern Italy, was chosen as a support material for its high-surface-area-for-unit-volume and relatively low price.

## Results

Pumice granules packed mini-columns of two different heights were colonized by a microbial consortium, isolated from a gasoline contaminated groundwater sample and enriched in the presence of toluene as the sole carbon and energy source. Afterwards, the mini-columns (henceforth named columns 1 and 2, see Methods for details) were continuously supplied with a mineral medium containing toluene as the target contaminant, and then sacrificed after 6- and 12-month operation, respectively.

Biofilms were characterized with three complementary approaches: observation by scanning electron microscopy, quantification of the attached biomass, and identification of the bacterial species, based on comparative 16S ribosomal DNA (rDNA) sequence analysis. Further, the biodegradative capability of the biofilm developed in column 2 was determined by measuring the efficiency of toluene removal, over a 12-month period.

### Scanning electron microscopy observation of the biofilm

**Figure 1:**
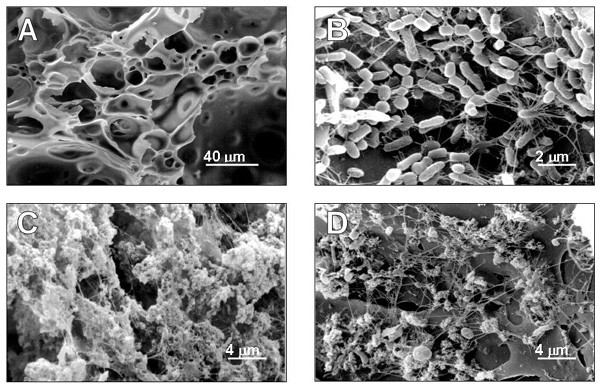
Scanning electron micrograph survey of pumice granules and biofilm development. Before colonisation (A) pumice granules are blank. After 6 month of operation (B), rod shaped cells cover the pumice surface. In the 12 month biofilm, an abundant exopolymeric matrix is visible on pumice granules both at the bottom (C) and top (D) of the column.

Scanning electron micrographs of Fig. 1 show the surface of the pumice support and the development and microscopic structure of the biofilm. Fig. 1A shows the macroporous structure of pumice stone before colonisation, Fig. 1B shows the surface of the pumice granules after 6 months of operation (column 1): rod shaped cells are evident. After 12 months operation (column 2), a thick layer of extracellular material covered the support, interspersed with a few single bacteria, both at the bottom (Fig. 1C) and the top (Fig. 1D) of the column.

### Quantification of the biomass adhering to pumice

Table 1 reports data on the determination of biomass adhering to pumice granules after 6- and 12-month operation (columns 1 and 2). In the case of column 1, the determination of biomass, carried out by burning and total protein assay (see Methods), showed that a biofilm of about 3 mg dry weight of cells per gram of pumice was established after 6-month run of the column with both methods. In the case of column 2, data obtained with the burning procedure clearly showed a significant increase in organic material (calculated as volatile solids, VS), both at the bottom and the top of the column. On the contrary, data based on total protein assay evidenced that the biomass increased only in the lower part of the column.

The discrepancy between data obtained with the two procedures could be attributed to the presence of a massive exopolymeric material in the 12 month-old biofilm, presumably of polysaccharidic nature and unavoidably weighed when the burning method is employed. The electron microscopy analysis suggests that this material is present both in the upper and lower part of the column; its production is linked to the biofilm maturation occurring in the second 6-month period. In fact, an abundant extracellular material covering the cells was evident by scanning electron microscopy of pumice granules sampled from both the upper and lower part of the column (Fig. 1). These considerations suggest that the protein assay could be a more reliable method to evaluate the actual amount of cells in the biofilm and to measure differences in biomass along the reactor. The different extent of colonization of the upper and lower part of column 2 could be presumably due to a higher nutrient supply at the bottom of the apparatus. Similarly, other authors [3,4] reported larger microbial populations at the inlet of the apparatus compared to the outlet, reflecting in this case a declining gradient of contaminant concentration along the reactor.

### Identification of the bacterial species

Identification of the species based on 16S rDNA comparative analysis revealed that in both biofilms (column 1 and 2) the majority (85%) of the attached cells was represented by *Rhodococcus erythropolis *(99% of identity), whereas *Pseudomonas marginalis *(98% of identity) represented only 10% of the entire consortium. On the contrary, in the enriched culture (the microbial consortium employed to start the development of the biofilm), *Pseudomonas marginalis *was the predominant species (86%) and *Rhodococcus erythropolis *was only 10% of the consortium. Apparently, adhesion to the pumice support promoted the growth of *Rhodococcus erythropolis*, modifying the initial ratio between the two species.

Less represented species in the three consortia examined (biofilms from column 1 and 2, as well as enriched culture) were identified as *Agrobacterium tumefaciens *(98% of identity), *Hydrogenophaga palleronii *(98% of identity), *Chryseobacterium scophtalum *(98% of identity), *Leucobacter komagatae *(98% of identity), *Brachybacterium articum *(96% of identity), *Beta proteobacterium *(98% of identity), *Microbacterium liquefaciens *(99% of identities) and *Acinetobacter sp. *(98% of identity).

### The biodegradative capability of the biofilm

**Figure 2:**
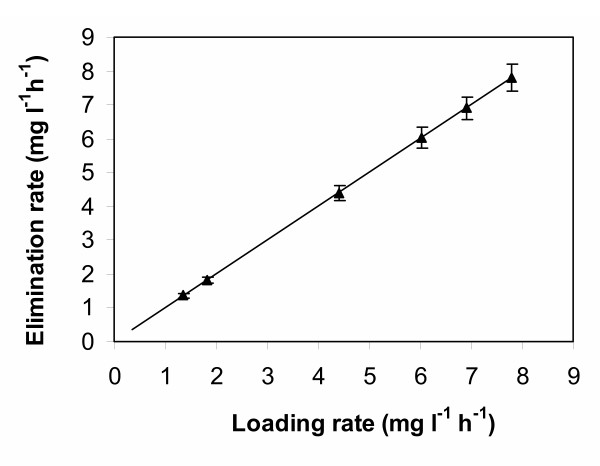
Toluene elimination rate of column 2 plotted as a function of toluene loading rate. Loading rate was increased over a 12-month period as described in the text.

The biodegradative capability of the biofilm developed on the pumice granules over 12 months was evaluated in terms of toluene removal efficiency (RE), calculated as the slope of the line obtained plotting the average toluene elimination rate (ER) *vs. *the loading rate (LR) applied to column 2 (Fig. 2). A linear relationship between the elimination rate and the applied loading rate was obtained over the whole experiment, implying that the column operated below its maximal degradation capacity. An overall toluene removal efficiency of 99 % was calculated from the slope of the line.

Toluene oxidation in the presence of nitrate as electron acceptor [5] may be postulated as the possible mechanism of toluene removal by the attached cells, since nitrate is present in the feeding medium and microaerophilic conditions presumably established along the column. Moreover, analysis of effluent revealed a nitrate concentration reduction (data not shown) consistent with the contribute of denitrifying bacteria in the biodegradation reaction.

## Conclusions

The results obtained in this work demonstrate that a structurally complex toluene-degrading biofilm developed on pumice granules, following their inoculation with a microbial consortium obtained by enrichment of toluene-contaminated water. In the biofilm, *Rhodococcus erythropolis *and *Pseudomonas marginalis *were the predominant species. Apparently, the two species, once attached to pumice stone, gave rise to a specialised community by the production of exopolymers functioning as biofilm stabilizers [6]. *Pseudomonas sp. *is the most studied single-species, biofilm-forming bacterium [6,7]; differently, the molecular details of biofilm formation by the Gram positive *Rhodococcus sp. *have not been investigated so far, though the genus *Rhodococcus *is present in biofilm reactors [8] for its broad metabolic diversity and ability to degrade hydrophobic pollutants [9].

Although the microbiological complexity of the consortium established on pumice deserves further investigation in order to clarify the specific role of each species and its contribution to toluene degradation, the results obtained in this work may be relevant for a final design of a biobarrier in which a cheap support as pumice is used for biofilm formation. In particular, the molecular analysis performed revealed the strong impact of immobilization on the structure of the toluene-degrading community, demonstrating the importance of a molecular approach to characterize microbial biofilms.

## Methods

A microbial consortium was obtained following enrichment of a gasoline contaminated groundwater sample and subsequently used to inoculate the packing material in the columns. The enrichment was carried out in 500 ml-flasks, adding 100 ml of groundwater to 100 ml of minimal salt medium (MSM), containing high sulphate and nitrate concentrations [10] supplemented with 20 mg l^-1 ^toluene (final concentration) as the sole carbon and energy source, sparged with air and incubated at 25°C for two weeks. After this acclimation period, 25% (v/v) of the culture was transferred into fresh MSM medium containing the same toluene concentration and incubated in the same conditions. To allow culture enrichment, the transfer was repeated 10 times, until a biomass (dry weight determination after centrifugation at 3000 *g *and washing of the collected cells) of 1.2 mg d. w. ml^-1 ^was achieved.

To study the biofilm development, two glass columns (2.5 cm in diameter and 1 or 3 cm in height), were packed with pumice granules (0.4–0.6 mm in size, average particle density 0.48 g ml^-1^), respectively previously washed with distilled water, autoclaved (120°C, 20 min) and then soaked in MSM medium containing 20 mg l^-1 ^toluene. The ratio between column and particle diameter was ~50 to reduce wall effects and preferred channelling; the column void volume was 60%. The columns were provided with teflon supports, tubes and connections to prevent abiotic removal of toluene.

**Figure 3:**
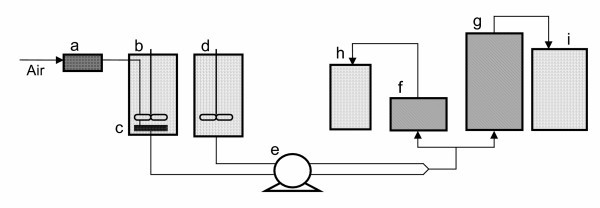
Schematic diagram of the mini-scale apparatus during continuous operation. *a*, air filter; *b*, feed tank; *c*, air diffuser; *d*, toluene tank; *e*, peristaltic pump; *f*, column 1; *g*, column 2; *h*, *i *effluent tanks.

For each column, the attachment of microbial cells to the support material was carried out by recirculating the enriched culture at a low flow rate (0.008 l h^-1^) for one week. After this period, the two columns, henceforth referred as column 1 (2.5 × 1 cm) and column 2 (2.5 × 3 cm), were continuously fed upwards at a flow rate (Q) of 0.026 ± 0.003 l h^-1^, with MSM plus toluene for 6 and 12 months, respectively, and then sacrificed. Operations were carried out at 25°C. MSM was continuously sparged with air and mixed with the solution containing toluene just before entering the columns. The set-up for the continuous operation of the two minicolumns is schematically presented in Fig. 3.

The inlet concentration of toluene (S_i_) was changed step-wise every 4 weeks, from an initial concentration of 0.77 ± 0.03 up to 4.42 ± 1.21 mg l^-1^, allowing two weeks to achieve culture acclimation before conducting the biodegradation assays in the following two weeks. A total of six values of toluene concentration in the inlet were established, being the highest inlet concentration achieved during the sixth month of operation; then, column 1 was sacrificed, and column 2 was fed with 4.42 ± 1.21 mg l^-1 ^toluene during the remaining 6 months, in order to permit biofilm maturation under constant feeding conditions.

In the case of column 2, values of toluene concentration in the inlet (S_i_) and in the outlet (S_e_) were employed to calculate toluene loading rate (LR) and elimination rate (ER) according to the following equations: ER = Q (S_i_-S_e_) / V and LR = S_i_·Q/V, where, Q is the inlet feed flow rate (0.026 l h^-1^) and V is the total volume (0.015 l) of the column, respectively.

### Toluene determination

Toluene concentration in the gas and liquid phase was determined by the headspace method on a gas chromatographic system (HP 6890 Series) equipped with flame ionization detector (FID), calculating the area of the chromatographic peaks. Toluene concentration values in the outlet of column 2 were the average of at least 10 determinations, carried out either after culture acclimation during the first 6-month operation, or every week during the following 6-month period, when column 2 was maintained at the highest LR. Neither evidence of abnormal biomass growth nor loss of mechanical properties of the supports were recorded during the entire time course of the experiment.

### Scanning electron microscopy analysis

To perform scanning electron microscopy analysis, pumice granules collected from both columns 1 and 2, together with not colonised granules were fixed with 2.5 % glutaraldehyde in 15 g l^-1 ^NaCl for two hours at 4°C, processed according to [2] and analysed with a scanning electron microscope (Cambridge 250 Mark3).

### Biomass determination

The biomass attached to the support was estimated as volatile solids (VS), after drying at 105°C and then burning at 600°C of the colonised pumice granules, collected from both columns. The difference in mass between the dried and the burned samples was the weight of VS (being ash equal to 8 ± 2% of the dry biomass). Biomass was also indirectly determined by total protein assay (Bio-Rad, Richmond, CA, USA) after detachment of the cells from the support by bead-beating in a homogenizer (Fast prep, BIO 101 Thermo Savant) and their lysis (60°C for 90 min with 0.6% w/v SDS), assuming that the average protein content in bacteria is about 50% of cell dry weight [11]. Since colonization of the top and the bottom parts of column 2 was considerably different to the naked eye, granules were sampled from both ends of the column and separately processed for all the determinations.

### Identification of the bacterial species

To identify the microbial species in the biofilm of columns 1 and 2, cells removed from the pumice granules by bead-beating (see above) were plated on TSA (Tryptone Soy Agar). An average of 100 colonies were isolated after serial dilutions from each consortium, and cultured individually in Tryptone Soy Broth (TSB). In parallel, to identify the microbial species present in the enriched culture employed to inoculate the packing material, an average of 100 colonies from the enriched culture were isolated on TSA and cultured individually in TSB.

Chromosomal DNA was extracted from cells of the isolated strains according to standard methods for bacteria [12] and used as template for PCR amplification performed with 30 cycles, each consisting of 30 s at 94°C for DNA denaturation; 40 s at 52°C for primer annealing, and 90 s at 72°C for elongation. Primers used in PCR reaction were P1 (5'-GCGGCGTGCCTAATACATGC) and P2 (5'-CACCTTCCGATACGGCTACC), annealing to nucleotides 40 to 59 and 1532 to 1513, respectively, of *B. subtilis rrnE*. These primers are normally utilized as universal primers for eubacteria. After PCR amplification, 1.5 Kbp 16S ribosomal DNA fragments were purified from agarose gels and sequenced. Ribosomal DNA gene sequences of the isolates where compared by BLAST program with those present in the DNA GenBank [13].

## Authors' contribution

DLA:carried out biofilm characterization. VM:carried out the identification of species and helped to draft the manuscript. PP: partecipated in the design of the study. VR: partecipated in the design of the study and setting up of laboratory apparatus. BA: performed toluene analysis. SP: partecipated in the setting up of laboratory apparatus. SR: partecipated in the design of the study and helped to draft the manuscript. DAE: conceived of the study, and partecipated in its design and coordination, drafted the manuscript. All authors read and approved the final manuscript.

## Figures and Tables

**Table 1:** Biomass adhering to pumice granules after 6- and 12- month operation

**Operation time **(months)	**Total biomass **(mg cells d.w. g^-1 ^pumice)	
	*Burning*	*Protein assay*

6	2.86	3.40
12 (top)	9.52	3.25
12 (bottom)	12.58	6.44
